# A study of the correlation among adolescents’ personal belief in a just world, resilience and teacher-student relationship: a second-order and mediation model

**DOI:** 10.3389/fpsyg.2026.1878362

**Published:** 2026-09-04

**Authors:** Zhuo Peng, Weijuan Zhang

**Affiliations:** 1 School of Foreign Studies, Shaoguan University, Shaoguan, China; 2 School of Education, South China Normal University, Guangzhou, China

**Keywords:** mediating roles, personal belief in a just world, resilience, structural equation modeling, teacher-student relationship

## Abstract

**Introduction:**

Clarifying the relation among adolescents’ personal belief in a just world (PBJW), resilience, and teacher-student relationship (TSR) can inform efforts to support youth mental health. Currently, many scholars probe into the pairwise relation among PBJW, resilience, and TSR, but investigating the relation among these 3 elements as a whole may provide more theoretical insights. In view of the current research states, this study analyzes the relation among adolescents’ PBJW, resilience, and TSR, and verifies the mediating roles of TSR between PBJW and resilience.

**Methods:**

This study employs questionnaire surveys and structural equation modeling to conduct the research. 1781 senior high school students in G province in China participated in this study. SPSS 25 and Amos 24 are employed to test reliability and validity of the survey, goodness-of-fit of the model, path relation, and Bootstrapping mediation effects, in order to verify 6 hypotheses proposed by this study.

**Results:**

Adolescents’ PBJW is positively associated with their resilience; the former is positively correlated with adolescents’ satisfaction with TSR and negatively related to their conflict with teachers; adolescents’ satisfaction with TSR and conflict with teachers are positively and negatively linked with their resilience, respectively; TSR could mediate the relation between adolescents’ PBJW and resilience.

**Conclusions:**

Relevant educational institutions can enhance adolescents’ resilience by reinforcing their PBJW and by establishing committees to oversee and enhance their TSR.

## 1 Introduction

Adolescence is a critical stage of individual growth, and adolescents are not yet physically and mentally mature. Their emotions are easily influenced by external factors, which make them more prone to psychological sub-health states such as anxiety and depression (Ho et al., 2018). According to Sappenfield et al. (2024), the rate of depression among adolescents was 8.4% in 2023, and the level of adolescents’ mental health is worrying. Better mental health is closely associated with higher resilience (Mesman et al., 2021), i.e., one important cause of adolescents’ mental problems is their low resilience in the face of adversity. Resilience is the capacity to overcome adversity and maintain psychological wellbeing (Wang and Zhang, 2024), and it can be assessed across 5 key dimensions: goal planning (GP), affect control (AC), positive thinking (PT), family support (FS) and help-seeking (HS) (Hu and Gan, 2008). Generally speaking, less resilient adolescents may find it difficult to adapt to difficult or challenging life experiences, and they tend to behave inflexibly (Geer and Zarchy, 2023). On the contrary, adolescents with strong resilience can efficiently exploit social supportive resources to alleviate the negative emotions arising from their perception of risk (Wang et al., 2022). In summary, adolescents’ resilience is a key psychological factor influencing their psychological development and quality of life (Wang and Zhang, 2024), and it is necessary to carry out investigation into adolescents’ resilience.

The personal belief in a just world (PBJW) is an important factor affecting adolescents’ mental health, which can make adolescents adhere to the belief in justice when they suffer from setbacks, so that they can live a normal and positive life, hence reducing the negative impact of setbacks on their psychology (Wu et al., 2011). According to *Belief in a Just World Theory* (*BJW Theory*), PBJW can make people less likely perceive themselves as the victims of discrimination and mitigate the subjective experiences of unfairness, therefore, PBJW can protect adolescents’ mental health, buffer against their stress and reduce their feelings of distress and threat (Dalbert, 2001). In other words, PBJW may enhance adolescents’ resilience against adversity. Meanwhile, PBJW can make people less suspicious of others and lead the former to believe that they will be fairly treated by others (Dalbert, 2001), thus, PBJW may strengthen adolescents’ relationship with teachers. In addition, according to *Developmental Resilience Theory*, individuals’ positive faith and outlooks on life are the protective factors for their resilience, which can enhance their adaptation to challenges and adversity in life, and hence, strengthening their resilience (Masten and Reed, 2002). Finally, according to *Attachment Theory*, the positive teacher-student relationship (TSR) can help alter students’ vulnerability and response to risk, and strengthen their resilience against adversity (Pianta and Steinberg, 1992). In sum, PBJW can enhance adolescents’ resilience and strengthen their ability to withstand setbacks (Wu et al., 2011), and TSR can affect the relation between PBJW and resilience. It can be seen that it is feasible to study the relation among adolescents’ PBJW, resilience, and TSR.

Currently, research on the relation among PBJW, resilience, and TSR mainly involves the relation between PBJW and resilience (Su and He, 2023), the bonds between resilience and TSR (Pianta and Steinberg, 1992), and the association between PBJW and TSR (Peter et al., 2012). Most of the relevant studies more or less concern *BJW Theory*, *Developmental Resilience Theory*, and *Attachment Theory*. However, these theories mainly deal with pairwise relation among PBJW, resilience, and TSR, and the literature remains theoretically fragmented. These pairwise approaches leave a critical void, i.e., it remains unclear how PBJW and TSR operate in concert to shape developmental outcomes in resilience. Specifically, the field lacks an integrative framework that specifies that the robust belief in a just world fosters the quality of TSR, which in turn may provide the support necessary for resilient adaptation. This framework moves beyond pairwise theorization by elucidating a sequential mechanism whereby TSR serves as the relational bridge between cognitive belief and adaptive capacity. By positioning TSR as such, the present study offers a theoretically grounded answer to how these 3 elements collaborate. In sum, investigating the relation among PBJW, resilience, and TSR by integrating these 3 theories could expand the theoretical boundaries of each, and clarify the factors that affect adolescents’ resilience, hence providing insights for enhancing adolescents’ resilience against adversity. Given these reasons, this study will analyze the relation among adolescents’ PBJW, resilience, and TSR.

## 2 Research on PBJW, resilience, and TSR

PBJW means that people believe that the world they live in is a just and orderly one, and that everything they obtain is what they deserve (Lerner and Miller, 1978). In other words, this is the belief that conveys good things should happen to good people while bad things should befall bad people (Galak and Chow, 2019). According to *BJW Theory*, PBJW can generate feelings of optimism and gratitude, which in turn regulate the relation between the PBJW and mental health, and the latter is relevant to an individual’s resilience (Jiang et al., 2016). In recent years, a large amount of research has further revealed that PBJW can alleviate the adverse impact of negative events, maintain an individual’s mental health, function as a psychological buffer, and enhance resilience (Wang and Meng, 2015). Tian et al. (2022) employ questionnaire surveys to demonstrate that young athletes’ PBJW is closely related to their resilience, and that the search for meaning in life and the presence of meaning in life play parallel mediating roles between young athletes’ PBJW and resilience, with the latter having a stronger mediating effect than the former. In addition, adolescents’ PBJW can enhance the promoting effect of their resilience on their subjective wellbeing (Su and He, 2023). In summary, as suggested by the *BJW Theory*, there may be correlation between adolescents’ PBJW and their resilience, but is this correlation significant? What factors can play mediating roles between the two? What are the characteristics of this correlation? These issues urgently need to be addressed.

TSR refers to the interpersonal relationship established between teachers and students through information exchange and communication in educational activities (Wang, 2012). On one hand, according to *BJW Theory*, PBJW can reduce adolescents’ suspicion of others and reinforce adolescents’ belief that they will receive fair treatment from others (Dalbert, 2001). In other words, adolescents’ PBJW may affect their judgment of TSR. The stronger their PBJW is, the more they believe that teachers will treat them fairly (Peter et al., 2012). Therefore, adolescents’ PBJW is closely related to their satisfaction (SAT) with and conflict (CON) in TSR. On the other hand, according to the *Attachment Theory*, positive TSR serves as a protective factor, which can mitigate students’ vulnerability to risk and enhance their resilience in the face of adversity (Pianta and Steinberg, 1992). Good TSR can enhance students’ resilience in difficult situations (Johnson, 2008) and effectively alleviate the negative effect of early adverse childhood experiences, abuse (Forster et al., 2017), and student bullying (Zhu and Teng, 2022). According to Zou et al. (2007), students’ SAT with TSR and CON with teachers are salient factors that reflect the quality of TSR. The degrees of SAT with and CON in TSR may influence students’ psychological functioning (Suldo et al., 2014), such as their resilience in difficult situations. In short, adolescents’ SAT with and CON in TSR are closely related to their PBJW and resilience. However, is PBJW simultaneously and significantly correlated with adolescents’ SAT with and CON in TSR, and their resilience? Do adolescents’ SAT with and CON in TSR play mediating roles between adolescents’ PBJW and resilience? These questions require further probing.

Resilience refers to an individual’s ability to overcome adversity and maintain mental health, it is a key psychological factor for an individual’s growth and success, and is extremely important for an individual’s psychological development and quality of life (Wang and Zhang, 2024). Resilience can be measured through 5 dimensions: GP, AC, PT, FS, and HS (Hu and Gan, 2008). Based on *Developmental Resilience Theory*, adolescents’ optimistic belief and positive perspectives on life can bolster their ability to cope with challenges and adversity in life (Masten and Reed, 2002). In other words, PBJW can promote adolescents’ ability to understand and interpret challenges and difficulties from more constructive perspectives, thereby enhancing their adaptability and resilience. Meanwhile, interpersonal relationship may have impact on adolescents’ resilience (Neustifter and Powell, 2015). Among the factors affecting resilience, support from others (including family, school, relatives, and friends of an individual) is the external protective factor (Marquez et al., 2023). Furthermore, compared to family background, school atmosphere has a more significant impact on students’ resilience (Jiang and Yao, 2022). School atmosphere involves the environment and situations created by the school, teachers, students, and other school staff (Dolati et al., 2021). The evaluation of school atmosphere concerns multiple aspects, such as TSR, school disciplinary climate, and student conduct (Pan, 2021). According to *Attachment Theory*, the positive TSR can be the key factor for strengthening adolescents’ resilience against adversity (Pianta and Steinberg, 1992). In summary, as suggested by *Developmental Resilience Theory* and *Attachment Theory*, there may be correlation among adolescents’ PBJW, TSR and resilience. However, not much is known about adolescents’ resilience currently (Scoloveno, 2015), and these 2 theories only deal with the pairwise correlation among adolescents’ PBJW, TSR and resilience. When these 3 aspects are integrated, are PBJW and TSR correlated with resilience simultaneously? and can the 2 theories be combined to jointly explain the correlation among PBJW, TSR and resilience? These questions deserve investigation.

In conclusion, firstly, according to *BJW Theory*, there is relation between PBJW and resilience, but few empirical studies have clarified the characteristics and the properties of this relation. Secondly, *BJW Theory* indicates that there may be some bonds between adolescents’ PBJW and TSR (adolescents’ SAT with and CON in TSR), but the salient degrees and the nature of these bonds still need further investigation. *Attachment Theory* implies that adolescents’ SAT with and CON in TSR may be linked with their resilience, however, not many empirical studies have quantified the magnitude of this relation and dealt with the mediating roles of adolescents’ TSR between their PBJW and resilience. Finally, *Developmental Resilience Theory* and *Attachment Theory* suggest that there may be correlation among adolescents’ PBJW, TSR and resilience, but limited empirical studies have probed into whether PBJW and TSR can be associated with resilience simultaneously, and not many studies have examined whether various relevant theories can be combined to jointly explain the correlation among PBJW, TSR and resilience. Given these reasons, this study investigates the relation between adolescents’ PBJW and resilience, and verify the mediating roles of TSR between adolescents’ PBJW and resilience. Hence, this study could expand the theoretical boundaries of *BJW Theory*, *Developmental Resilience Theory* and *Attachment Theory* by integrating them, and provide insights for improving adolescents’ resilience. Accordingly, 6 hypotheses are established for the study.

*H1*: Adolescents’ PBJW is associated with their resilience.

*H2*: Adolescents’ PBJW is correlated with their SAT with TSR.

*H3*: Adolescents’ SAT with TSR is related to their resilience.

*H4*: Adolescents’ PBJW is linked with their CON with teachers.

*H5*: Adolescents’ CON with teachers is associated with their resilience.

*H6*: Adolescents’ SAT with and CON in TSR mediate the relation between their PBJW and resilience.

**Figure 1:**
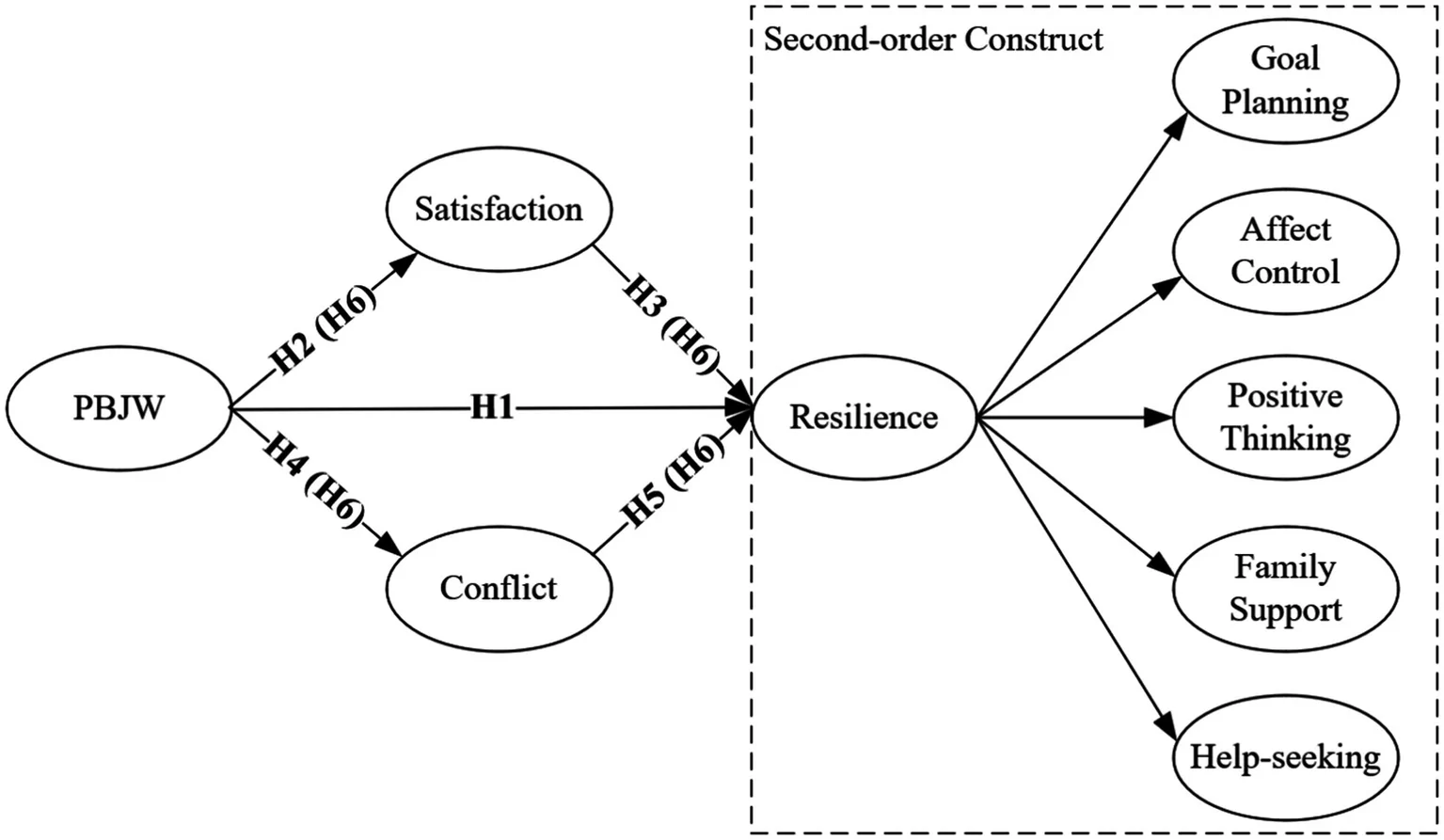
The theoretical model.

The theoretical model is constructed in Figure 1.

## 3 Research design

### 3.1 Research subjects

The research subjects are the students in a senior high school in G province in China. The reason for choosing the students from this school as survey subjects is that one of the researchers is a teacher at this school, and the familiarity between the teacher and students makes it easier to conduct the survey there. Additionally, the researcher is well-acquainted with these students, most of whom are known for their ability to strictly follow teachers’ instructions and complete classroom tasks. This ensures that their response in the survey is genuine and valid. Moreover, the students are diverse in terms of family background and economic condition, which allows them to represent a broad range of opinions in this survey.

Concerning the potential power asymmetry inherent in the researcher-teacher dual role, this study implements the following measures to minimize social desirability bias and coercion in the survey. Firstly, the survey is introduced as an independent research project unrelated to course assessment. Secondly, complete anonymity of the students is guaranteed. Thirdly, participation is entirely voluntary, and students are reminded that their decision to participate or withdraw will not affect their course grades.

This study was reviewed and approved by the College-Level Science Ethics Committee at South China Normal University, and formal approval was granted on October 21, 2025. Additionally, participants and their guardians were informed that involvement was voluntary, that participants could withdraw at any time without academic penalty, and that all responses would be kept strictly anonymous and confidential.

This study conducts paper-based questionnaire surveys on the students, and a total of 1944 questionnaires are collected, of which 163 are excluded due to patterned or incomplete responding, resulting in 1781 valid questionnaires, with a validity rate of 91.62%. Table 1 displays the demographic features of the respondents.

**Table 1:** Sample features.

Indicators	Items	Freq.	%
Respondent sources	TSFYHS	384	21.56
	TSSYHS	405	22.74
	ASFYHS	429	24.09
	ASSYHS	563	31.61
Gender	Male	829	46.50
	Female	952	53.50
Boarding students	Yes	1,433	80.46
	No	348	19.54
The only child of a family	Yes	241	13.53
	No	1,540	86.47
Left-behind adolescents	Yes	139	7.80
	No	1,642	92.20
A single-parent family	Yes	127	7.13
	No	1,654	92.87
Experiencing recent negative events	Yes	93	5.22
	No	1,688	94.78
Family’s economic condition	Very good	96	5.39
	Average	1,529	85.85
	Moderately difficult	156	8.76
Age	14 years old	4	0.22
	15 years old	309	17.35
	16 years old	877	49.24
	17 years old	568	31.89
	18 years old	22	1.24
	19 years old	1	0.06

TSFYHS, TSSYHS, ASFYHS, and ASSYHS represent top students in the first year of high school, top students in the second year of high school, average students in the first year of high school, and average students in the second year of high school, respectively.

### 3.2 Research tools

#### 3.2.1 PBJW Scale

This study employs the PBJW scale developed by Dalbert (1999) and translated and adapted by Dong and Lin (2011). This Chinese scale is utilized to measure the factor of adolescents’ personal belief in a just world. The scale consists of 7 items. In this study, 1 item with factor loading less than 0.600 is removed in the exploratory factor analysis (EFA) (see Supplementary Table S1). Additionally, 2 more items are removed based on the modification index output in Amos 24. The pre-deletion and post-deletion goodness-of-fit indices for the eight-factor model (PBJW, SAT, CON, GP, AC, PT, FS, and HS) with correlated factors in confirmatory factor analysis (CFA) are presented in Supplementary Table S3. Finally, there retain 4 items with high reliability and validity in PBJW scale as shown in Table 2. Moreover, the scale utilizes a 5-point positive scoring system, with scores ranging from 1 to 5 corresponding to 5 options: “completely disagree,” “mostly disagree,” “uncertain,” “mostly agree,” and “completely agree.”

**Table 2:** Reliability and validity analyses.

Dimension	Item	Unstd.	SE	*Z*	*p*	Std.	Cronbach’s *α*	CR	AVE
PBJW	PBJW1	1.000				0.761	0.844	0.846	0.579
	PBJW2	0.975	0.032	30.066	***	0.740			
	PBJW3	1.060	0.032	33.290	***	0.827			
	PBJW4	0.901	0.031	28.901	***	0.712			
SAT	SAT1	1.000				0.827	0.800	0.826	0.618
	SAT2	1.126	0.030	37.295	***	0.897			
	SAT3	0.863	0.033	26.071	***	0.605			
CON	CON1	1.000				0.704	0.786	0.789	0.557
	CON2	1.153	0.043	27.034	***	0.838			
	CON3	0.901	0.036	24.877	***	0.688			
GP	GP1	1.000				0.708	0.764	0.768	0.527
	GP2	0.893	0.038	23.516	***	0.659			
	GP3	1.085	0.041	26.153	***	0.803			
AC	AC1	1.000				0.658	0.763	0.772	0.532
	AC2	1.291	0.052	24.597	***	0.812			
	AC3	1.183	0.050	23.496	***	0.710			
PT	PT1	1.000				0.522	0.737	0.756	0.516
	PT2	1.740	0.087	19.892	***	0.786			
	PT3	1.621	0.081	19.931	***	0.811			
FS	FS1	1.000				0.734	0.804	0.807	0.583
	FS2	1.081	0.037	29.205	***	0.833			
	FS3	1.058	0.039	27.042	***	0.718			
HS	HS1	1.000				0.781	0.783	0.788	0.557
	HS2	1.005	0.034	29.321	***	0.811			
	HS3	0.793	0.032	24.634	***	0.634			

***indicates *p* < 0.001.

#### 3.2.2 Resilience Scale

This study exploits the adolescent resilience scale developed by Hu and Gan (2008). This Chinese scale is composed of 5 dimensions: GP, AC, PT, FS, and HS, which are the 5 factors of measuring adolescents’ resilience. Among these 5 factors, GP concerns making plans, adhering to goals and concentrating on solving problems in adversity; AC involves controlling and adjusting emotional fluctuations and pessimism in adversity; PT deals with the dialectical and optimistic attitudes towards adversity; FS pertains to family members’ respectful and supportive attitudes towards adolescents; HS entails obtaining help or venting emotions through meaningful interpersonal interaction (Hu and Gan, 2008). In this study, 2 items from GP, 1 item from AC, 1 item from PT, and 2 items from FS are eliminated in the EFA due to factor loadings less than 0.600 (see Supplementary Table S1). Furthermore, according to the modification index output in Amos 24, 2 items from AC, 1 item from FS, and 3 items from HS are deleted because of their negative influence on the goodness-of-fit indices of the eight-factor model with correlated factors in CFA (see Supplementary Table S3). Finally, this study retains 3 items in each of the above 5 dimensions, respectively. The adapted scale is presented in Supplementary Table S2. This scale adopts a 5-point scoring system. Except for items of AC1, AC2, AC3, FS1, FS2, FS3, HS1, HS2, and HS3, which are scored in reverse, the options “completely disagree,” “mostly disagree,” “uncertain,” “mostly agree,” and “completely agree” in the remaining items are scored from 1 to 5 points, respectively.

#### 3.2.3 TSR Scale

This study exploits 2 sub-scales (SAT and CON) of TSR Chinese scale developed by Zou et al. (2007) for the reason that these sub-scales display relatively high reliability and validity as shown in Table 2. These 2 sub-scales are employed to measure the 2 factors of adolescents’ SAT with and CON in TSR. This study discards 1 item from SAT and 3 items from CON in the EFA due to their factor loadings less than 0.600, meanwhile, this study deletes 1 item from SAT scale because this item does not load on a common factor with other SAT items (see Supplementary Table S1). Additionally, according to the modification index output in Amos 24, 1 more item from CON is removed due to its negative influence on the goodness-of-fit indices of the eight-factor model with correlated factors in CFA (see Supplementary Table S3). Eventually, each of the sub-scales of SAT and CON retains 3 items as shown in Supplementary Table S2. Meanwhile, these sub-scales adopt a 5-point scoring system, with 1 to 5 points corresponding to 5 options: “completely disagree,” “mostly disagree,” “uncertain,” “mostly agree,” and “completely agree.” In summary, certain items are eliminated from the scales in this study based on scientific rationale. While this may narrow the construct coverage, it ensures the reliability and validity of the remaining items and enhances goodness-of-fit of the model.

### 3.3 Data analyses

This study analyzes the scale data with the help of SPSS 25 and Amos 24. The Pearson correlation analyses (2-tailed) of the means of the PBJW, SAT, CON, GP, AC, PT, FS, and HS are presented in Table 3.

**Table 3:** Correlation analyses.

Constructs	Means	SD	1	2	3	4	5	6	7	8
1. PBJW	3.524	0.739	1.000							
2. SAT	3.608	0.779	0.501^**^	1.000						
3. CON	1.995	0.809	−0.291^**^	−0.388^**^	1.000					
4. GP	3.267	0.877	0.360^**^	0.360^**^	−0.111^**^	1.000				
5. AC	2.775	1.007	0.264^**^	0.198^**^	−0.211^**^	0.310^**^	1.000			
6. PT	3.591	0.805	0.341^**^	0.353^**^	−0.167^**^	0.474^**^	0.231^**^	1.000		
7. FS	3.494	0.939	0.266^**^	0.224^**^	−0.349^**^	0.184^**^	0.348^**^	0.179^**^	1.000	
8. HS	2.933	0.998	0.237^**^	0.182^**^	−0.210^**^	0.231^**^	0.460^**^	0.134^**^	0.417^**^	1.000

^***^indicates *p* < 0.001, ^**^indicates *p* < 0.01.

Firstly, the study utilizes Harman’s single-factor testing and common method factor modeling to check whether there is common method bias in the measurement data. Secondly, reliability and validity testing, as well as discriminant validity testing, are conducted to examine the internal consistency, the convergent validity and discriminability of dimensions in the measurement model of this study. Thirdly, model fit testing is performed to assess whether the second-order model and the overall structural model have good fits with the observed data. Fourthly, path relation testing is carried out to evaluate whether hypotheses H1 ~ H5 in this study are valid. Finally, Bootstrapping mediating testing is employed to evaluate the mediating roles of TSR between adolescents’ PBJW and their resilience, and verify hypothesis H6.

## 4 Research results

### 4.1 Common method bias testing

This study utilizes Harman’s single-factor testing to assess common method bias in the measurement data. The study conducts an EFA of all items in the scales, and the results show that there are 8 factors with eigenvalues greater than 1, and the first unrotated factor accounts for 26.483% of the variation, which is less than the criterion of 40%. Therefore, the result suggests that common method bias is unlikely to be a serious concern (Podsakoff et al., 2003).

To further evaluate common method bias, a common method factor (CMF) was added to the measurement model of 8 factors, with all manifest indicators constrained to load equally onto the method factor (Podsakoff et al., 2003). Table 4 displays the goodness-of-fit indices of the measurement model with and without the CMF.

**Table 4:** Model fit comparison before and after adding a common method factor.

Treatments	*χ^2^*	*df*	CFI	RMSEA	SRMR	CMF Avg. loading
Without CMF	929.614	247	0.961	0.039	0.037	–
With CMF	924.261	246	0.961	0.039	0.037	0.174
*Δ*	5.353	1	0.000	0.000	0.000	–

According to Table 4, although the chi-square difference test is significant (Δ*χ^2^* = 5.353, Δ*df* = 1, *p* = 0.021), the chi-square statistic is highly sensitive to sample size and tends to produce significant values in large samples (Bentler and Bonett, 1980; Hair et al., 2019; Kline, 2016). Additionally, the change in fit indices is negligible (ΔCFI = 0.000, ΔRMSEA = 0.000, ΔSRMR = 0.000), and the average standardized loading of the CMF is 0.174, suggesting that method variance accounted for only a small fraction of the variance in the indicators. In sum, these results indicate that common method bias is unlikely to be a serious threat in this study.

### 4.2 Reliability and validity testing

With the help of SPSS 25 and Amos 24, this study conducts CFA, and the results are shown in Table 2.

According to Table 2, the Cronbach’s α and composite reliability (CR) in all dimensions are greater than 0.7, which means that the items in each dimension have high consistency and good reliability. Meanwhile, most of the standardized factor loadings are greater than 0.7. Although some standardized factor loadings are less than 0.7, all the *Z* values are greater than 1.96, and all the *p*-values are less than 0.001, in addition, all the average variances extracted (AVE) are greater than 0.5, which indicates that the convergent validity of each dimension is passable.

By employing Amos 24, this study carries out the discriminant validity test, and the results are shown in Table 5.

**Table 5:** Discriminant validity test.

Dimensions	HS	FS	PT	AC	GP	CON	SAT	PBJW
HS	**0.746**							
FS	0.526	**0.764**						
PT	0.172	0.212	**0.718**					
AC	0.567	0.415	0.287	**0.729**				
GP	0.296	0.236	0.584	0.381	**0.726**			
CON	−0.276	−0.449	−0.176	−0.268	−0.149	**0.746**		
SAT	0.232	0.271	0.375	0.238	0.400	−0.492	**0.786**	
PBJW	0.291	0.322	0.391	0.317	0.445	−0.362	0.539	**0.761**

In Table 5, the bold values on the diagonal of the table are the square roots of the AVE. and these values are all greater than the correlation coefficients of their respective horizontal and vertical dimensions, which indicates that the dimensions of the measurement model have good discriminant validity.

### 4.3 Model fit testing

After passing the reliability and validity testing, the structural model of this study undergoes model fit testing. With the help of Amos 24, this study calculates the goodness-of-fit indices of second-order construct of Resilience, the results are presented in Table 6.

**Table 6:** Goodness-of-fit indices of second-order construct.

Resilience	*χ^2^*	*df*	*χ^2^*/*df*	GFI	AGFI	IFI	CFI	TLI	RMSEA	SRMR
First-order one-factor model	4614.542	90.000	51.273	0.691	0.588	0.510	0.509	0.427	0.168	0.129
First-order five-factor model with uncorrelated factors	1809.457	90.000	20.105	0.866	0.822	0.814	0.813	0.782	0.104	0.192
First-order five-factor model with correlated factors	374.067	80.000	4.676	0.973	0.959	0.968	0.968	0.958	0.045	0.031
Second-order factor model	685.111	85.000	8.060	0.953	0.933	0.935	0.935	0.920	0.063	0.071
Ref.	/	/	<5	>0.9	>0.9	>0.9	>0.9	>0.9	<0.08	<0.08

Table 6 shows that the reference value of *χ^2^*/*df* is less than 5. As for the construct of Resilience, the *χ^2^*/*df* value of the first-order five-factor model with correlated factors (i.e., the first-order model formed by GP, AC, PT, FS, and HS with correlation) is 4.676 (*χ^2^*/*df*<5), and the values of GFI, AGFI, IFI, CFI, and TLI are greater than 0.9, what’s more, the values of RMSEA and SRMR are less than 0.08 (Meng, 2024). These results indicate the first-order five-factor model with correlated factors has a good fit with the observed data. In addition, the *χ^2^*/*df* value of second-order factor model is 8.060 (*χ^2^*/*df*>5), but the values of GFI, AGFI, IFI, CFI, and TLI of the model are greater than 0.9, and the values of RMSEA and SRMR are less than 0.08 (Meng, 2024). Since the chi-square statistic tends to produce a significant value in a large sample, the *χ^2^*/*df* ratio is rendered an unreliable standalone criterion (Bentler and Bonett, 1980; Hair et al., 2019; Kline, 2016), and the fit indices (GFI, AGFI, IFI, CFI, TLI, RMSEA, and SRMR) that are less susceptible to sample-size inflation should be prioritized as more important criteria for model fit assessment. In summary, the second-order factor model shows a passable level of fit. Furthermore, the target coefficient (the ratio of chi-square of the first-order model to the chi-square of the second-order factor model) indicates the extent to which the second-order factor model accounts for the first-order model with correlated factors, and this coefficient ranges from 0 to 1, the higher the coefficient, the more the second-order factor model can represent the first-order factor model (Marsh and Hocevar, 1985). The target coefficient of the second-order factor model of Resilience is 374.067/685.111 = 0.546, therefore, the accountability of the second-order factor model for the first-order factor model is passable.

By exploiting Amos 24, this study calculates the goodness-of-fit indices of the overall structural model, which is presented in Table 7.

**Table 7:** Goodness-of-fit indices of the overall structural model.

Overall model
Ref.	*χ* ^2^	*df*	*χ*^2^/*df*	GFI	AGFI	IFI	CFI	TLI	RMSEA	SRMR
Indices	1643.618/	265.000	6.202	0.928	0.912	0.922	0.922	0.911	0.054	0.070
Values	/	/	<5	>0.9	>0.9	>0.9	>0.9	>0.9	<0.08	<0.08

As can be seen from Table 7, the *χ^2^*/*df* value of the overall model is slightly greater than 5. As the chi-square statistic tends to produce a significant value in a large sample (Bentler and Bonett, 1980; Hair et al., 2019; Kline, 2016), the alternative fit indices should be the more important evidence for assessing the model. The values of GFI, AGFI, IFI, CFI, and TLI of the overall model are greater than 0.9, and the values of RMSEA and SRMR are less than 0.08 (Meng, 2024), therefore, the goodness-of-fit of the overall model is passable.

### 4.4 Hypothesis testing for the model

**Figure 2:**
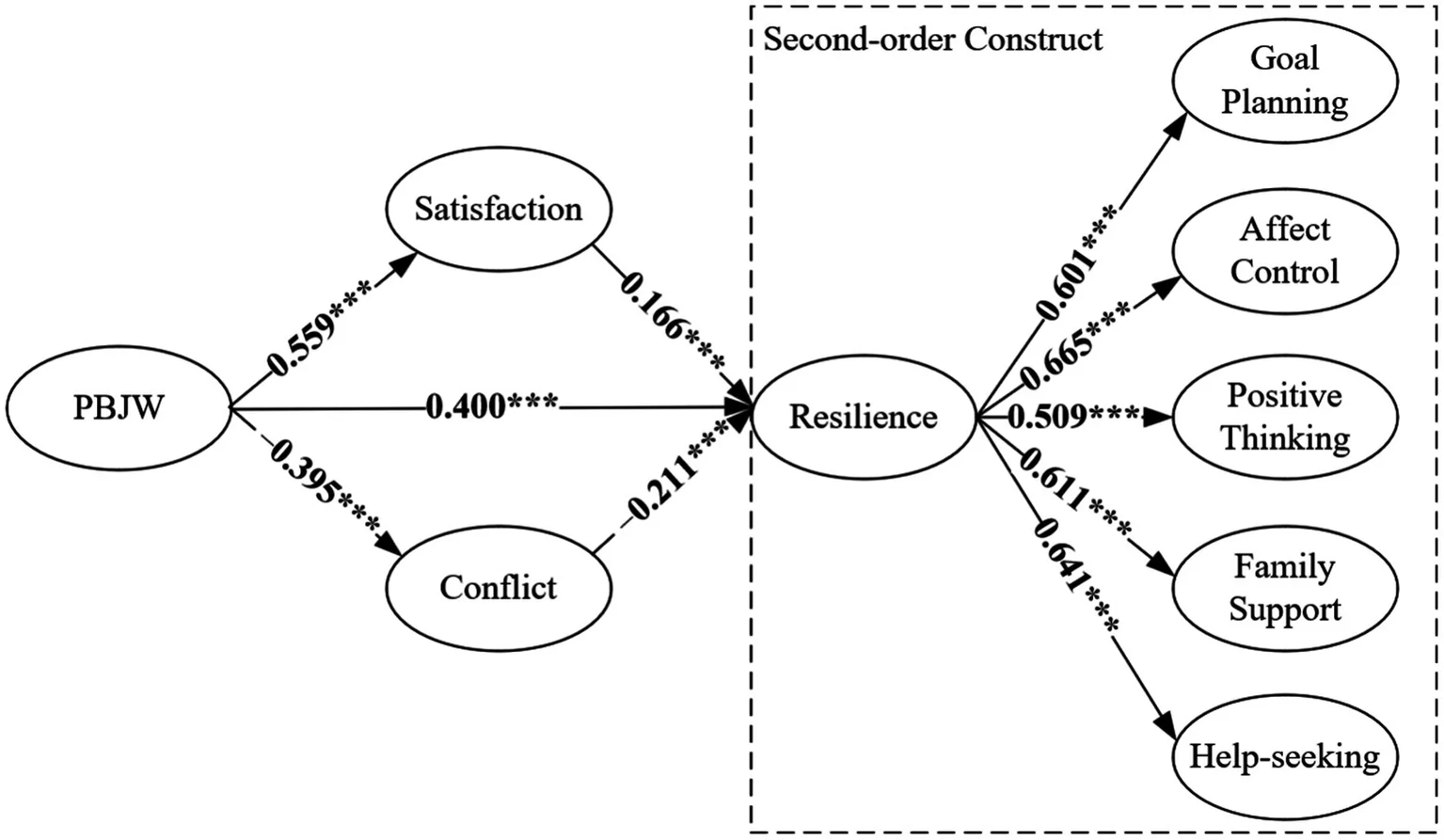
Path relation testing of the structural model. ***indicates *p* < 0.001.

Since the overall structural model of this study has an adequate fit with the observed data, the study can proceed to further examine the path relation within the model, and the results are shown in Figure 2 and Table 8.

**Table 8:** Path relation testing.

Hypotheses	Paths	Unstd.	SE	*Z*	*p*	Std.	Testing results
H1	PBJW → Resilience	0.299	0.033	9.119	***	0.400	Support
H2	PBJW → SAT	0.639	0.034	18.988	***	0.559	Support
H3	SAT → Resilience	0.109	0.023	4.669	***	0.166	Support
H4	PBJW → CON	−0.405	0.031	−12.897	***	−0.395	Support
H5	CON → Resilience	−0.154	0.024	−6.299	***	−0.211	Support

^***^indicates *p* < 0.001.

According to Figure 2, the *p-*values of the paths from resilience to GP, AC, PT, FS, and HS are less than 0.001, which prove the validity of these paths, i.e., adolescents’ resilience can be effectively measured by the dimensions of GP, AC, PT, FS, and HS. These dimensions embody adolescents’ resilience from the perspectives of cognition, emotion, behavior, and environment (Hu and Gan, 2008).

According to Table 8, the standardized path coefficients of H1, H2, H3, H4, and H5 are 0.400, 0.559, 0.166, −0.395, and −0.211, respectively. All the absolute values of *Z* scores of the paths are greater than 1.96 and all the *p-*values of the paths are less than 0.001, they verify the 5 hypotheses in this study are reasonable. Specifically, firstly, adolescents’ PBJW is positively associated with their resilience; secondly, their PBJW is positively correlated with their SAT with TSR; thirdly, their SAT with TSR is positively linked with their resilience; fourthly, their PBJW is negatively related to their CON with teachers; finally, their CON with teachers is negatively correlated with their resilience.

### 4.5 Mediating effect testing

By employing Amos 24, this study conducts Bootstrapping mediating effect testing of the structural model, and the results are presented in Table 9.

**Table 9:** Analyses of the mediating effects.

Path relation	Point estimate	Product of coefficients		Bootstrapping 1,000 times 95% CI			
				Bias-corrected		Percentile	
		SE	*Z*	Lower	Upper	Lower	Upper
Indirect effects							
PBJW → SAT → Resilience	0.069	0.022	3.136	0.030	0.119	0.030	0.118
PBJW → CON → Resilience	0.062	0.012	5.167	0.041	0.088	0.037	0.086
PBJW → SAT (CON) → Resilience (IE)	0.132	0.022	6.000	0.094	0.179	0.093	0.177
Direct effect							
PBJW → Resilience (DE)	0.299	0.046	6.500	0.211	0.391	0.214	0.398
Total effect							
Total effect (TE)	0.431	0.052	8.288	0.323	0.533	0.325	0.537
Percentage of indirect effect							
Percentage (IE/TE)	0.306	0.047	6.511	0.222	0.403	0.222	0.404
Ratio of indirect effect to direct effect							
Ratio (IE/DE)	0.441	0.101	4.366	0.285	0.676	0.286	0.678

According to Table 9, the bias-corrected Bootstrapping 95% confidence intervals for the direct effect and the indirect effect of adolescents’ PBJW on resilience do not contain 0, which indicates that PBJW may not only have a significant direct effect on resilience, but also have a robust indirect effect on resilience through the mediating factors of SAT with and CON in TSR. In other words, the hypothesis H6 in this study is verified. The direct effect (0.299) and the indirect effect (0.132) account for 69.37% and 30.63% of the total effect (0.431), respectively. Meanwhile, the ratio of the indirect effect to the direct effect is 0.441. In summary, TSR could play significant mediating roles between adolescents’ PBJW and resilience.

## 5 Discussion

This study analyzes the relation among adolescents’ PBJW, TSR and resilience, and verifies the mediating roles of TSR between PBJW and resilience. The research results in the current sample indicate that adolescents’ PBJW is positively associated with resilience, which is similar to the views of Su and He (2023) and of Wang and Meng (2015). Meanwhile, the results of this study also show that TSR could significantly predict adolescents’ resilience, which is similar to the findings of Jiang and Yao (2022) and of Neustifter and Powell (2015). In addition, this study suggests that adolescents’ PBJW is significantly correlated with TSR, which is consistent with the views of Peter et al. (2012). More specifically, the current sample discloses that adolescents’ PBJW is positively associated with their SAT with TSR and negatively related to their CON with teachers, and adolescents’ SAT with TSR is positively correlated with their resilience and their CON with teachers is negatively linked with their resilience. Therefore, the mediating roles of TSR between PBJW and resilience are validated.

Based on *BJW Theory*, *Developmental Resilience Theory*, and *Attachment Theory*, it is reasonable for this study to examine the relation among PBJW, TSR, and resilience simultaneously. When the 3 theories are applied separately, they can only explain the pairwise relation among PBJW, TSR, and resilience. However, when these theories are integrated, the new fusion theory can explain the relation among PBJW, TSR, and resilience as a whole. Specifically, *BJW Theory* provides a theoretical foundation for relation between PBJW and resilience, and between PBJW and TSR. *Attachment Theory* underpins the relation between TSR and resilience, in addition, *Developmental Resilience Theory* reinforces the link between PBJW and resilience. Therefore, through the mediation of *Attachment Theory*, *BJW Theory* and *Developmental Resilience Theory* can explain how PBJW is associated with TSR, and subsequently correlated with resilience. In other words, the theoretical boundaries of the 3 theories are expanded to account for the relation among PBJW, TSR, and resilience.

According to *BJW Theory*, PBJW could buffer against adolescents’ stress and distress (Dalbert, 2001), and as suggested by *Developmental Resilience Theory*, adolescents’ positive faith and outlooks on life are the protective factors for their resilience (Masten and Reed, 2002), hence, PBJW could enhance adolescents’ resilience against adversity. Adolescents’ PBJW could make them believe that they live in a just world, and good behavior will receive good reward (Lerner and Miller, 1978). Therefore, this belief could inspire optimism and a sense of gratitude in adolescents (Jiang et al., 2016). Optimistic mindset enables adolescents to focus on positive aspects of things, thereby encouraging them to face various adversity and setbacks with positive attitudes. This may allow them to generate lasting and strong motivation in the process of overcoming difficulties, thus displaying strong resilience.

According to *Attachment Theory*, positive TSR could help students become less vulnerable to risk and more resilient in the face of adversity (Pianta and Steinberg, 1992). Furthermore, by integrating *BJW Theory* and *Developmental Resilience Theory*, it is reasonable that adolescents’ TSR plays mediating roles between their PBJW and resilience. Adolescents’ PBJW may lead them to believe that others will treat them fairly, and even when faced with seemingly unfair treatment, they may maintain faith that justice will ultimately be served to them. Therefore, adolescents’ PBJW may predict the way they handle TSR. It may encourage adolescents to firmly believe that teachers will treat them fairly (Peter et al., 2012), which could make them view the teachers’ action with optimism and positivity, thereby increasing their SAT with TSR and reducing the likelihood of CON between adolescents and teachers. In addition, good TSR is closely correlated with students’ resilience in difficult situations (Johnson, 2008). The more the adolescents’ SAT with TSR, the more likely they are to feel their teachers’ support and care, the more capable they are of generating positive emotions to cope with various academic and life challenges, thereby subtly enhancing their resilience. However, when adolescents have CON with teachers, they may experience psychological pressure in dealing with interpersonal relationship, feel a lack of authoritative support and assistance in their personal growth, and may gradually lose the will and confidence to cope with academic and life challenges, thereby, their resilience may decline. In summary, TSR could play mediating roles between adolescents’ PBJW and resilience.

The results of this study show adolescents’ resilience could be measured from 5 dimensions: GP, AC, PT, FS, and HS. These dimensions involve the efficacy of cognitive, emotional, behavioral, and environmental elements in enabling adolescents to overcome adversity and attain positive adaptation (Hu and Gan, 2008), which encompass the internal and external elements that may predict adolescents’ resilience. Meanwhile, adolescents’ PBJW is positively associated with their resilience. When facing difficulties and challenges, adolescents with strong PBJW are more inclined to believe that the world is fair, and thus actively respond to adversity and improve their resilience. This means that cultivating and enhancing adolescents’ PBJW may help them strengthen resilience and better cope with various challenges in life. In addition, TSR could play mediating roles between adolescents’ PBJW and their resilience. This indicates that in order to enhance adolescents’ resilience, it may be not only necessary to strengthen their inner PBJW, but also to improve their SAT with TSR and reduce their CON with teachers, so as to increase their external protective resources and help them establish healthy psychological mechanisms. However, this mediating effect is partial, because adolescents’ PBJW could be directly linked with their resilience. The direct and indirect effects accounted for 69.37% and 30.63% of the total effect, respectively. Therefore, although TSR may enhance or weaken the relation between adolescents’ PBJW and resilience, it does not play a decisive role in this relation.

Given the correlation among adolescents’ PBJW, resilience, and TSR verified in this study, if we are eager to improve adolescents’ mental health, the following measures could be taken: firstly, relevant educational institutions could create good campus atmosphere, actively promote positive moral paradigms, and strengthen adolescents’ PBJW. Secondly, since TSR may act as an important mediator between PBJW and resilience, educational institutions and families should enhance communication with adolescents to promptly understand their relationship with teachers. It is essential for educational institutions to strengthen mental health education and counseling mechanisms, enabling adolescents to freely and timely share their interpersonal CON and psychological concerns. Meanwhile, the establishment of parent-teacher co-education committees, school open days, and parent reception days, along with the organization of parent–child social practice activities, will provide parents with more opportunities to understand the development of their children’s interpersonal relationship at school, thereby enhancing the intervention of both families and educational institutions in adolescents’ interpersonal relationship. Thirdly, given that TSR is closely correlated with adolescents’ resilience, teachers should increase their love and care for adolescents, especially offering more help and support to those in difficult situations, thereby building good TSR and promoting the improvement of adolescents’ resilience.

The most salient theoretical contribution of this study lies in revealing the sequential mechanism through which cognitive belief translates into adaptive developmental outcomes. Instead of regarding PBJW and TSR as parallel factors, this study suggests that PBJW operates as a cognitive precursor that drives a relational process. Adolescents who maintain robust belief in a just world are likely to form high-quality bonds with teachers. In turn, these bonds provide the emotional scaffolding necessary for resilient adaptation. This finding extends *BJW Theory* by displaying that the adaptive value of justice belief is not limited to direct psychological benefits, rather, PBJW exerts its protective function through relational channels, a process that has been theoretically implied but empirically underexamined in prior literature.

Although this study conducts detailed analyses of adolescents’ PBJW, resilience, TSR, and their correlation, there are still limitations of this study, such as the limited sample drawn from a single educational institution, which may not fully represent adolescents from different social classes and in different areas. Consequently, the findings should be interpreted as preliminary and context-bound. Another notable limitation of this study concerns the use of factor loadings and goodness-of-fit indices to trim nearly half of the items in the adolescent resilience scale. While this procedure is common practice in scale validation, the construct validity evidence for the trimmed resilience scale may not be directly comparable to that of the original full scale. The trimmed model should be cross-validated on a new independent sample in future. In addition, this study only carries out cross-sectional analyses, and might need further validation through longitudinal analyses. Therefore, future research can further expand the sample size and explore the psychological development characteristics of adolescents across a wider range of areas and over a specific period, so as to provide more comprehensive theoretical foundations and practical guidance for adolescents’ mental health regulation.

## 6 Conclusion

This study investigates the correlation among adolescents’ PBJW, resilience, and TSR, and verifies the mediating roles of TSR between PBJW and resilience. The 6 hypotheses proposed in this study have been validated with the current sample: firstly, adolescents’ PBJW is positively associated with their resilience; secondly, their PBJW is positively correlated with their SAT with TSR; thirdly, their SAT with TSR is positively related to their resilience; fourthly, their PBJW is negatively linked with their CON with teachers; fifthly, their CON with teachers is negatively correlated with their resilience; finally, TSR could play mediating roles between adolescents’ PBJW and their resilience. This study could clarify the relation among adolescents’ PBJW, resilience, and TSR, expand the theoretical boundaries of the research on these 3 elements, and provide insights for decreasing adolescents’ psychological problems caused by low resilience.

## Data Availability

The original contributions presented in the study are included in the article/Supplementary material, further inquiries can be directed to the corresponding author.

## Glossary

AC: Affect control
AGFI: Adjusted goodness-of-fit index
ASFYHS: Average students in the first year of high school
ASSYHS: Average students in the second year of high school
AVE: Average variances extracted
BJW: Belief in a just world
*χ^2^*: Chi-square
CFA: Confirmatory factor analysis
CFI: Comparative fit index
CMF: Common method factor
CON: Conflict
CR: Composite reliability
DE: Direct effect
*df*: Degrees of freedom
FS: Family support
GFI: Goodness-of-fit index
GP: Goal planning
H: Hypothesis
HS: Help-seeking
IE: Indirect effect
IFI: Incremental fit index
PBJW: Personal belief in a just world
PT: Positive thinking
Ref: Reference
RMSEA: Root mean square error of approximation
SAT: Satisfaction
SE: Standard error
SRMR: Standardized root mean square residual
Std: Standardized estimate
TE: Total effect
TLI: Tucker-Lewis index
TSFYHS: Top students in the first year of high school
TSR: Teacher-student relationship
TSSYHS: Top students in the second year of high school
Unstd: Unstandardized estimate
