# Supplementary material for: A study of the correlation among adolescents’ personal belief in a just world, resilience and teacher-student relationship: a second-order and mediation model
Source: Front Psychol. 2026 Sep 4;17:1878362. doi: 10.3389/fpsyg.2026.1878362 (PMC13585978; doi:10.3389/fpsyg.2026.1878362)
Supplement: Supplementary file 1 [file Supplementary_file_1.docx]

**Supplementary Material**

Table S1 Original Item Sets and Rotated Factor Loadings in EFA

| Scales | Items | Factor 1 | Factor 2 | Factor 3 | Factor 4 | Factor 5 | Factor 6 | Factor 7 | Factor 8 | Factor 9 |
| --- | --- | --- | --- | --- | --- | --- | --- | --- | --- | --- |
| PBJW | Overall, events in my life are just. (总的来说，在我生活中发生的事是公正的。) | 0.792 |  |  |  |  |  |  |  |  |
|  | I believe that most of the things that happen in my life are fair. (我相信在我生活中发生的大部分事情是公正的。) | 0.764 |  |  |  |  |  |  |  |  |
|  | I am usually treated fairly. (我通常受到了公正的对待。) | 0.760 |  |  |  |  |  |  |  |  |
|  | I believe that I usually get what I deserve. (我相信我通常得到了我所应得的。) | 0.730 |  |  |  |  |  |  |  |  |
|  | In my life injustice is the exception rather than the rule. (在我生活中发生的不公正事件是偶然的，而不是必然的。) | 0.712 |  |  |  |  |  |  |  |  |
|  | I think that important decisions that are made concerning me are usually just. (我认为凡是涉及到我的所作的重大决定通常是公正的。) | 0.691 |  |  |  |  |  |  |  |  |
|  | To a large extent, I believe that what happens to me is what I deserve. (在很大程度上，我相信在我身上发生的事都是我应得的。) | 0.555 |  |  |  |  |  |  |  |  |
| CON | I feel that my teachers always punish and criticize me. (我觉得老师总是惩罚和批评我。) |  | 0.780 |  |  |  |  |  |  |  |
|  | I think my teachers are unfair to me. (我觉得老师对我不公平。) |  | 0.743 |  |  |  |  |  |  |  |
|  | My teacher and I always seem to struggle with each other. (老师和我似乎总是在相互斗争。) |  | 0.730 |  |  |  |  |  |  |  |
|  | My teachers and I often disagree with each other and sometimes we even argue. (我和老师经常看法不一致，有时还会争吵。) |  | 0.721 |  |  |  |  |  |  |  |
|  | I find it hard to get along with teachers. (与老师相处使我感到很费劲。) |  | 0.591 |  |  |  |  |  |  |  |
|  | Even when I feel sad or wronged, my teachers rarely show concern for me. (即使在我难过或委屈的时候，老师也很少关心我。) |  | 0.566 |  |  |  |  |  |  |  |
|  | My teachers seldom pay attention to me in classes. (在班上老师很少注意我。) |  | 0.408 |  |  |  |  |  |  |  |
| AC | I always find it difficult to control my unpleasant emotions. (我总是很难控制自己的不愉快情绪。) |  |  | 0.762 |  |  |  |  |  |  |
|  | Failure always makes me feel discouraged. (失败总是让我感到气馁。) |  |  | 0.746 |  |  |  |  |  |  |
|  | Failures and setbacks will make me doubt my abilities. (失败和挫折会让我怀疑自己的能力。) |  |  | 0.709 |  |  |  |  |  |  |
|  | It usually takes me a long time to forget unpleasant things. (我一般要过很久才能忘记不愉快的事情。) |  |  | 0.657 |  |  |  |  |  |  |
|  | I have many mood swings and am prone to ups and downs. (我情绪波动很大，容易大起大落。) |  |  | 0.644 |  |  |  |  |  |  |
|  | I can adjust my emotions very well in a short time. (我能很好地在短时间内调整情绪。) |  |  | 0.493 |  |  |  |  |  |  |
| HS | When I'm in a bad mood, I'm not willing to talk to others about it. (心情不好也不愿意和别人说。) |  |  |  | 0.723 |  |  |  |  |  |
|  | I am used to keeping things to myself rather than confiding in others. (我习惯把事情憋在心里而不是找人倾诉。) |  |  |  | 0.720 |  |  |  |  |  |
|  | When I have difficulties, I take the initiative to talk to someone about them. (我有困难时会主动找人倾诉。) |  |  |  | 0.709 |  |  |  |  |  |
|  | I always can’t find a suitable person to talk to when encountering unpleasant things. (当我遇到不愉快的事情时，总找不到合适的倾诉对象。) |  |  |  | 0.672 |  |  |  |  |  |
|  | I have a peer friend whom I can talk to about my difficulties. (我有一个同龄的朋友，可以把困难说给他（她）听。) |  |  |  | 0.649 |  |  |  |  |  |
|  | I don’t know who to turn to when I am in trouble. (当我遇到困难时，我不知道应该去找谁。) |  |  |  | 0.628 |  |  |  |  |  |
| GP | I usually set a plan or solution when faced with difficulties. (面临困难，我一般会定一个计划或解决方案。) |  |  |  |  | 0.716 |  |  |  |  |
|  | I set goals for myself to push myself forward. (我会为自己设定目标，以推动自己前进。) |  |  |  |  | 0.710 |  |  |  |  |
|  | I have clear goals in life. (我有明确的生活目标。) |  |  |  |  | 0.696 |  |  |  |  |
|  | I will concentrate all my energy when facing difficulties. (面对困难时，我会集中自己的全部精力。) |  |  |  |  | 0.599 |  |  |  |  |
|  | I generally become more mature and experienced after experiencing setbacks. (经历挫折后我一般会更加成熟有经验。) |  |  |  |  | 0.512 |  | 0.461 |  |  |
| FS | My parents always like to interfere with my ideas. (父母总是喜欢干涉我的想法。) |  |  |  |  |  | 0.735 |  |  |  |
|  | My parents respect my opinions very much. (父母很尊重我的意见。) |  |  |  |  |  | 0.724 |  |  |  |
|  | My parents always lack confidence in and emotional support for me. (父母总是对我缺乏信心和精神上的支持。) |  |  |  |  |  | 0.715 |  |  |  |
|  | At home, mostly no one listens to what I say. (在家里，我说什么总是没人听。) |  |  |  |  |  | 0.666 |  |  |  |
|  | My parents never scold me harshly. (父母从来不苛责我。) |  |  |  |  |  | 0.580 |  |  |  |
|  | My parents always encourage me to give it my all. (父母总是鼓励我全力以赴。) |  |  |  |  |  | 0.533 |  |  |  |
| PT | Adversity can sometimes help growth. (逆境有时候对成长是一种帮助。) |  |  |  |  |  |  | 0.783 |  |  |
|  | I believe that adversity has a motivating effect on me. (我认为逆境对我有激励作用。) |  |  |  |  |  |  | 0.681 |  |  |
|  | I feel that the process of things helps me grow more than the result. (我觉得与结果相比，事情的过程更能助人成长。) |  |  |  |  |  |  | 0.635 |  |  |
|  | I believe there is a positive aspect to everything. (我觉得任何事情都有积极的一面。) |  |  |  |  |  |  | 0.531 |  |  |
| SAT | Overall, I am satisfied with my relationship with my teachers. (总的来说，我很满意自己和老师的关系。) |  |  |  |  |  |  |  | 0.763 |  |
|  | The present relationship between my teachers and me is exactly what I want. (目前我和老师的关系正是我所希望的。) |  |  |  |  |  |  |  | 0.723 |  |
|  | My communication with my teachers makes me feel confident and accomplished. (与老师的交往使我感到自信和有成就。) |  |  |  |  |  |  |  | 0.625 |  |
|  | I treasure the relationship between my teachers and me. (我很珍惜我和老师之间的关系。) |  |  |  |  |  |  |  | 0.508 |  |
|  | I hope to improve my relationship with my teachers. (我希望改善我和老师的关系。) |  |  |  |  |  |  |  |  | 0.793 |

Table S2 Rotated Factor Loadings in EFA after Item Deletion for Scales

| Scales | Items | Factor 1 | Factor 2 | Factor 3 | Factor 4 | Factor 5 | Factor 6 | Factor 7 | Factor 8 |
| --- | --- | --- | --- | --- | --- | --- | --- | --- | --- |
| PBJW | PBJW3. I believe that most of the things that happen in my life are fair. (我相信在我生活中发生的大部分事情是公正的。) | 0.816 |  |  |  |  |  |  |  |
|  | PBJW2. In my life injustice is the exception rather than the rule. (在我生活中发生的不公正事件是偶然的，而不是必然的。) | 0.787 |  |  |  |  |  |  |  |
|  | PBJW1. Overall, events in my life are just. (总的来说，在我生活中发生的事是公正的。) | 0.785 |  |  |  |  |  |  |  |
|  | PBJW4. I think that important decisions that are made concerning me are usually just. (我认为凡是涉及到我的所作的重大决定通常是公正的。) | 0.727 |  |  |  |  |  |  |  |
| CON | CON1. My teacher and I always seem to struggle with each other. (老师和我似乎总是在相互斗争。) |  | 0.806 |  |  |  |  |  |  |
|  | CON2. I think my teachers are unfair to me. (我觉得老师对我不公平。) |  | 0.794 |  |  |  |  |  |  |
|  | CON3. I feel that my teachers always punish and criticize me. (我觉得老师总是惩罚和批评我。) |  | 0.786 |  |  |  |  |  |  |
| FS | FS1. My parents always like to interfere with my ideas. (父母总是喜欢干涉我的想法。) |  |  | 0.810 |  |  |  |  |  |
|  | FS3. My parents always lack confidence in and emotional support for me. (父母总是对我缺乏信心和精神上的支持。) |  |  | 0.803 |  |  |  |  |  |
|  | FS2. At home, mostly no one listens to what I say. (在家里，我说什么总是没人听。) |  |  | 0.790 |  |  |  |  |  |
| HS | HS1. I always can’t find a suitable person to talk to when encountering unpleasant things. (当我遇到不愉快的事情时，总找不到合适的倾诉对象。) |  |  |  | 0.826 |  |  |  |  |
|  | HS2. I don’t know who to turn to when I am in trouble. (当我遇到困难时，我不知道应该去找谁。) |  |  |  | 0.790 |  |  |  |  |
|  | HS3. I am used to keeping things to myself rather than confiding in others. (我习惯把事情憋在心里而不是找人倾诉。) |  |  |  | 0.748 |  |  |  |  |
| SAT | SAT2. Overall, I am satisfied with my relationship with my teachers. (总的来说，我很满意自己和老师的关系。) |  |  |  |  | 0.846 |  |  |  |
|  | SAT1. The present relationship between my teachers and me is exactly what I want. (目前我和老师的关系正是我所希望的。) |  |  |  |  | 0.819 |  |  |  |
|  | SAT3. My communication with my teachers makes me feel confident and accomplished. (与老师的交往使我感到自信和有成就。) |  |  |  |  | 0.653 |  |  |  |
| AC | AC2. I always find it difficult to control my unpleasant emotions. (我总是很难控制自己的不愉快情绪。) |  |  |  |  |  | 0.833 |  |  |
|  | AC1. Failure always makes me feel discouraged. (失败总是让我感到气馁。) |  |  |  |  |  | 0.775 |  |  |
|  | AC3. I have many mood swings and am prone to ups and downs. (我情绪波动很大，容易大起大落。) |  |  |  |  |  | 0.703 |  |  |
| GP | GP3. I set goals for myself to push myself forward. (我会为自己设定目标，以推动自己前进。) |  |  |  |  |  |  | 0.790 |  |
|  | GP1. I have clear goals in life. (我有明确的生活目标。) |  |  |  |  |  |  | 0.786 |  |
|  | GP2. I usually set a plan or solution when faced with difficulties. (面临困难，我一般会定一个计划或解决方案。) |  |  |  |  |  |  | 0.734 |  |
| PT | PT3. Adversity can sometimes help growth. (逆境有时候对成长是一种帮助。) |  |  |  |  |  |  |  | 0.851 |
|  | PT2. I believe that adversity has a motivating effect on me. (我认为逆境对我有激励作用。) |  |  |  |  |  |  |  | 0.778 |
|  | PT1. I feel that the process of things helps me grow more than the result. (我觉得与结果相比，事情的过程更能助人成长。) |  |  |  |  |  |  |  | 0.643 |

Table S3 Comparison of Goodness-of-Fit Indices between Pre-Deletion and Post-Deletion of Items in Measurement Models

| Eight-Factor Model with Correlated Factors | Treatments | *χ*^2^ | *df* | *χ*^2^/*df* | GFI | AGFI | IFI | CFI | TLI | RMSEA | SRMR |
| --- | --- | --- | --- | --- | --- | --- | --- | --- | --- | --- | --- |
|  | Pre-Deletion of Items | 6125.544 | 961 | 6.374 | 0.847 | 0.828 | 0.851 | 0.851 | 0.839 | 0.055 | 0.060 |
|  | Post-Deletion of Items | 929.614 | 247 | 3.764 | 0.959 | 0.947 | 0.961 | 0.961 | 0.953 | 0.039 | 0.037 |
|  | Ref. | / | / | <5 | >0.9 | >0.9 | >0.9 | >0.9 | >0.9 | <0.08 | <0.08 |
